# Effects of Dietary Grape Seed Meal Bioactive Compounds on the Colonic Microbiota of Weaned Piglets With Dextran Sodium Sulfate-Induced Colitis Used as an Inflammatory Model

**DOI:** 10.3389/fvets.2020.00031

**Published:** 2020-02-26

**Authors:** Iulian A. Grosu, Gina C. Pistol, Daniela E. Marin, Ana Cişmileanu, Laurenţiu M. Palade, Ionelia Ţăranu

**Affiliations:** Laboratory of Animal Biology, National Institute for Research and Development for Biology and Animal Nutrition, Balotesti, Romania

**Keywords:** inflammatory bowel diseases, colitis, piglet, grape seed meal, dextran sodium sulfate, microbiota

## Abstract

Microbiota affects host health and plays an important role in dysbiosis. The study examined the effect of diet including grape seed meal (GSM) with its mixture of bioactive compounds on the large intestine microbiota and short-chain fatty acid synthesis in weaned piglets treated with dextran sodium sulfate (DSS) as a model for inflammatory bowel diseases. Twenty-two piglets were included in four experimental groups based on their diet: control, DSS (1 g/kg/b.w.+control diet), GSM (8% grape seed meal inclusion in control diet), and DSS+GSM (1 g/kg/b.w., 8% grape seed meal in control diet). After 30 days, the colon content was isolated and used for microbiota sequencing on an Illumina MiSeq platform. QIIME 1.9.1 pipeline was used to process the raw sequences. Both GSM and DSS alone and in combination affected the diversity indices and *Firmicutes:Bacteroidetes* ratio, with significantly higher values in the DSS-afflicted piglets for *Proteobacteria* phylum, *Roseburia, Megasphera* and *CF231* genus, and lower values for *Lactobacillus*. GSM with high-fiber, polyphenol and polyunsaturated fatty acid (PUFA) content increased the production of butyrate and isobutyrate, stimulated the growth of beneficial genera like *Prevotella* and *Megasphaera*, while countering the relative abundance of *Roseburia*, reducing it to half of the DSS value and contributing to the management of the DSS effects.

## Introduction

The intestinal inflammatory bowel disease (IBD) affects the life quality of a large number of people and is a significant problem for public health (1–3). Although it is now known that IBDs are symptoms of an unbalanced inflammatory response between commensal microflora, pathogens, and the host immune system (4), the precise nature of the intestinal microbiota perturbation and the resulting effects remains to be identified. Most of the risk factors implicated in the development of IBD, including diet, stress and anti-inflammatory drugs, can also perturb the commensal component of the microbiota (5, 6). While the microbiota of healthy hosts shows little shifts in time, the gut microbiota of IBD affecting hosts is not stable. Dysbiosis in IBD do not just change the populations of different microbiota species but is also associated with perturbations of microbial metabolites, like short-chain fatty acids (SCFAs), which can further affect the host (7). There is growing interest to manipulate the gut microbiota for preventative and therapeutic purposes.

In recent years, alternative remedies were studied as promising therapy for IBD, some of the most important ones being the use of natural bioactive compounds with high anti-inflammatory activity such as polyphenols, polyunsaturated fatty acids (PUFAs). Also, SCFAs (acetate, n-propionate, and n-butyrate), which are solely produced by gut microbiota and have shown to ameliorate the disease effects. Studies have demonstrated that dietary polyphenols such as flavonols, stilbenoids, and anthocyanins, or chlorogenic acid derived from tomatoes (8, 9) and blueberries (10) had positive effects in animals with dextran sodium sulfate (DSS)-induced colitis. For example, Scarano et al. (8) demonstrated that mice with DSS-induced colitis fed with tomato diet rich in polyphenols were characterized by a significant “re-shaping” of the gut microbiota in terms of composition when compared to the DSS group, as indicated by a significant increase of the ratio *Bacteroidetes: Firmicutes* as compared with the control. Also, dietary blueberries or broccoli influenced the composition and metabolism of the cecal microbiota and colon morphology in a mice model of IBD (10). Other polyphenol sources found to re-shape the microbiota composition in mice model of IBD are grape seed extract (11) and curcumin (12). In the study of Wang et al. (11), grape seed extract rich in polyphenols increase the abundance of non-pathogenic bacteria in the gut, contributing to the improvement of gut function and IBD symptoms. Also, these dietary bioactive compounds impact the colon positively by affecting the transit time and the production of SCFAs that further affect the pH and enhance the gut barrier properties along with also a protective effect on the colonic mucosa (13). PUFAs have shown to modulate the microbiota dynamics in animal models of IBD. Constantini et al. (14) have demonstrated that ω-3 PUFAs lead to microbiota enrichment with more beneficial bacterial strains. The eicosapentaenoic acid-free fatty acid diet counteracts the DSS-dependent dysbioses of the gut microbiota, facilitating the recovery of a health-promoting layout of the gut microbial ecosystem in mice (15).

Various animal models were used for more than two decades to investigate the pathogenesis and etiology of human IBD to gain indispensable insights into morphological, metabolic, and microbiota changes as well as on other factors associated with the evolution of IBD but also for therapeutic evaluation. The models of chemically induced IBD have used different animal species (mice, rats, and rabbits) (5, 16, 17). Mouse have been considered the most suitable animal model for the relative analogy to human intestine in terms of immune response and inflammatory genes (18).

Recently, pig held an essential place as an animal model due to the similarities they share with humans in terms of gastrointestinal morphology and physiology, which makes them suitable for human studies (7, 19, 20). In particular, pigs are considered to be an excellent large-animal model to study intestinal inflammation in humans (21). Additionally, the pig microbiome is also comparable to humans, facilitating the examination of the relationship between microbial communities, diet, and intestinal health (22). Nutritional interventions, such as ω-3 PUFAs administration, proved to modulate the inflammation and contributed to delaying the onset of experimental DSS-induced IBD in pigs (23).

Using Illumina high-throughput sequencing of the 16S rRNA gene, we aimed in the present study to investigate the capacity of the grape seed meal (GSM) as a dietary rich source of bioactive compounds (polyphenols, ω-6 fatty acids, fibers, etc.) to alleviate the DSS-induced alterations of bacterial diversity and the microbial community composition at the phylum and lower taxonomical levels. Active molecules derived from grape or grape by-products and their effect on IBD have been investigated in the mouse model, but mostly as individual components. In the present study, we investigated the effect of the entire complex of bioactive compounds from grape seed by-product, taken as example the Mediterranean diets that through the diversity of ingredients (fresh vegetables, fruits, nuts, fish, and olive oil) and their high concentration in different bioactive nutrients provided promising results by alleviating IBD symptoms and increasing microbiota diversity. To our knowledge, this is the first study that evaluates the capacity of GSM to modulate the microbiota of DSS-treated piglets as well as the correlations between microbiota composition and the production of colonic SCFAs.

## Materials and Methods

### Animals and Experimental Treatments

Twenty-two TOPIGS-40 hybrid healthy weaned piglets (9.13 ± 0.03 kg average body weight) were individually ear-tagged and randomly assigned to four experimental groups (5–6 piglets/group) based on their initial body weight as follows: (1) Control; (2) DSS; (3) GSM; (4) DSS+GSM.

Control and DSS groups were fed a standard diet based on maize and soybean meal. GSM and DSS+GSM groups were fed the control diet, including 8% dried GSM without interfering with the nutritional requirements of weaning piglets, performance, size, and digestibility. The diets were formulated to meet all nutritional requirements for post-weaning piglets (24) as described by (25). Ingredients and chemical composition of the diets are presented in Tables 1A–C. The GSM was provided by a local commercial company (S.C. OLEOMET-SA S.R.L., Bucharest, Romania).

**Table 1A:** Composition and nutrient content of experimental diets (%).

**Ingredients (%)**	**Control diet**	**GSM diet**
Corn	67.47	58.5
Soybean meal	19	18
Gluten	4	4
Milk replacer	5	5
Soya oil	–	2
L Lysine	0.4	0.4
DL Methionine	0.1	0.15
Monocalcium phosphate	1.46	1.33
Feed grade limestone	1.37	1.42
Salt	0.1	0.1
Choline premix	0.1	0.1
Vitamin mineral premix^a^	1.0	1.0
Grape seed meal	–	8
**Analyzed composition**		
Crude protein (%)	18.42	18.21
Fat (%)	3.03	3.19
Cellulose (%)	3.12	5.8
Lysine (%)	1.2	1.2
Methionine +Cysteine (%)	0.72	0.72
Calcium (%)	0.90	0.90
Phosphorus (%)	0.65	0.65
Metabolizable energy (ME, kcal/kg)	3,248	3,178

a *Vitamin–mineral premix/kg diet: (0–18 days): 10,000 UI vit. A; 2,000 vit. D; 30 UI vit. E; 2 mg vit. K; 1.96 mg vit. B1; 3.84 mg vit. B2; 14.85 mg pantothenic ac.; 19.2 mg nicotinic ac.; 2.94 mg vit. B6; 0.98 mg folic ac.; 0.03 mg vit. B12; 0.06 biotin; 24.5 mg vit. C; 40.3 mg Mn; 100 mg Fe; 100 mg Cu; 100 mg Zn; 0.38 I; 0.23 mg Se*.

**Table 1B:** Antioxidant activity and polyphenols content of experimental diets.

**Item**	**Control diet**	**GSM diet**
DPPH (μM TRE/g sample)	206.89	966.35
Total polyphenols (mg GAE/100 g)	382.93	897.15
**Polyphenols composition (μg/mL extract catechin equivalent)**		
Hydroxycinnamic acids	318.11	362.25
Flavonols	0	311.12
Isoflavonoids	85.24	122.42
Anthocyanins	0	187.65

**Table 1C:** Composition in fatty acids of experimental diets.

**Polyunsaturated fatty acid content**	**Control diet**	**GSM diet**
Total PUFA (g/100 g total FAME)	47.58	52.01
Total ω-3 FA (g/100 g total FAME)	2.20	1.45
Total ω-6 FA (g/100 g total FAME)	45.38	50.56
ω-6/ω-3 ratio	20.61	34.88

DSS (dextran sulfate 40 sodium salt, MW = 36–50 kDa, Carl Roth GmbH, Germany, 1 g/kg body weight) was orally administered to DSS and DSS+GSM experimental groups for 5 consecutive days. Two cycles of DSS treatment (days 1–5 and 21–26 of the experiment) were used to induce chronic intestinal inflammation in piglets.

All piglets from each experimental group were housed in a large box (a box/group) and every group included mixed sexes. The body weight was recorded at the beginning (day 0) and at the end of the feeding experiment (day 30) for each animal; the feed intake was recorded daily/pen/group. Piglets were fed the experimental diet for 30 days and had free access to food and water all along the experimental period. After 30 days, the piglets were sacrificed, and content from the descending colon was collected from each animal, which was immediately stored at −80°C until further use.

During the whole experimental period, the stool cosinstency was assessed daily. Piglets did not receive veterinary treatments for diarrhea. For each experimental group, the diarrhea incidence was calculated with the following formula adapted after (26): (total number of diarrhea-affected piglets/total number of experimental piglets) × 100%.

### Chemical Characterization of the Diets

Feed samples of control and experimental diets were analyzed for nutrient content, dry matter, crude protein, crude fat, crude fiber, and ash according to the International Standard Organization methods [SR ISO 6496/2001, Standardized Bulletin (2010) http://www.asro.ro].

Total polyphenol content was measured and identification of different classes of polyphenols and PUFAs of the diets was carried out by Folin-Ciocalteu reaction, HPLC-DAD-MS, and gas chromatography as described by Taranu et al. (25, 27). Diet antioxidant activity was measured in terms of hydrogen donating or radical scavenging ability, using the stable radical, DPPH, as described previously (28).

### Sampling and 16s rRNA Sequencing

Microbial genetic material was extracted from 200 ml colonic content samples using the QIAGEN mini Stool Kit (Qiagen, Dusseldorf, Germany) as described by Grosu et al. (29). The DNA integrity and concentration were verified on gel electrophoresis and Nanodrop Spectrophotometer. The library formation and sequencing of the 16S rRNA gene were carried out using a MiSeq® Reagent Kit V3-V4 on a MiSeq-Illumina® platform using the 300PE approach by BMR Genomics (Padova, Italy).

### Microbiota Bioinformatics and Statistical Analysis

The FastQ raw data sequences resulting from the Illumina platform sequencing were further processed using an open reference OTU (operational taxonomic unit) strategy in QIIME (v1.9.1) (30) with default settings. The bacterial OTUs were generating using the UCLUST function with a *de novo* protocol of 97% similarity threshold. Taxonomy was assigned to the resulting representative sequences by comparing against the Greengenes database v13_8 with the help of the UCLUST method, selecting the similarity threshold of 90%. OTUs with a relative abundance of ≤0.005% were removed and were Chimera checked in QIIME with the Blast fragments approach. In order to remove sampling depth heterogeneity, a rarefaction with a cutoff of 23,946, which represents the lowest number of reads from a sample, was performed.

Alpha (within-sample) diversity (estimated with Chao1, observed_otus, PD_whole_tree) and beta (between-sample) diversity (DPCoA) indices were generated using the phylogeny-based unweighted and weighted UniFrac metrics. An OTU-based phylogenetic tree was also generated using FastTree method inside QIIME. A heatmap was also built around the OTU table of the species that were found above a 0.005% relative abundance.

### GC Method for SCFAs in Pig Feces

SCFAs (acetic, propionic, butyric and valeric acids) were quantified in water extracts of pig's colon content sample by gas chromatography. Briefly, colon samples were mixed with distilled water in a proportion of 1:2 (w:v), centrifuged at 12,000 g for 25 min and diluted 1:2 with distilled water. A sample volume of 1 μL from the centrifuged extract was injected under split mode into a gas chromatograph (Varian, 430-GC) equipped with a capillary column Elite-FFAP with a length of 30 m, an inner diameter of 320 μm, and a film thickness of 0.25 μm (Perkin Elmer, USA). The carrier gas was hydrogen; flow, 1.5 mL/per min. The injector was set at 250°C, and the split rate was 1:40. The flame ionization detector (FID) was set to 200°C, and the column oven was set to 110°C. The oven temperature was increased to 170°C at a rate of 12°C/min, where it was held for 9.5 min. The analysis time was 10 min. The sample concentration was calculated referring to a standard commercial mixture of volatile fatty acids (CRM46975, Supelco, USA). Results were expressed as μmol/g for total SCFAs and as a percentage for individual SCFA.

### Statistical Analysis

The internal statistical method used by QIIME in determining significance between sample groups was performed using the ANOSIM statistical method, a non-parametric method; the significance is determined through permutations. Statistical significance of difference like comparisons between effects was performed under XLstat software package (http://www.xlstat.com) using two-way analysis of variance (ANOVA). The model effects were DSS, GSM, and their interaction (DSS × GSM), in order to evaluate the overall treatment effect. Values of *p* < 0.05 indicated statistically significant differences among the different comparisons. The results are presented as mean ± SEM. The heatmap built on the OTU table for a relative abundance above 0.005% with clustering for OTU ID and treatment was also constructed using XLstat. Additionally, effect sizes were reported for the model effects as described by Lakens (31). Eta squared (η^2^) measures the proportion of the total variance in a dependent variable that is associated with the membership of different groups defined by an independent variable. Omega squared (ω^2^) is an estimate of how much variance in the response variables are accounted for by the explanatory variables.

## Results

### Diet Composition

The chemical composition of control and GSM diet is presented in Tables 1A–C. GSM experimental diet had an increased content of fibers (cellulose) compared to the control diet (5.80 vs. 3.12%, respectively, Table 1A). Also, the GSM diet had a higher concentration of polyphenols and an increased antioxidant activity compared to that of the control diet (Table 1B). GSM used in the present study had a total polyphenol content of 5567.22 mg GAE/100 g sample (data not shown). HPLC-DAD–MS analysis showed that GSM was rich in flavonoids (catechins, epicatechins, and procyanidins), the highest concentration being observed for caffeoylquinic acid (57.36 mg/100 g), ferulic acid derivate (34.43 mg/100 g), and dicaffeoylquinic acid (28.85 mg/100 g) (data not shown). Also, our results showed the presence of the antioxidant activity (DPPH) in GSM (5054.71 μM TRE).

The composition in PUFA of the GSM diet was 52.01/100 g of fatty acid methyl esters (FAME) (Table 1C) of which the highest proportion was registered for ω-6 fatty acids (50.56 g/100 g FAME) compared to the control diet (47.58 total PUFA and 45.38 g ω-6 fatty acids/100 g FAME). Notably, the ratio of ω-6/ω-3 PUFAs was increased in the GSM diet compared to the control diet (34.88 vs. 20.61, Table 1C). The gas chromatography analysis showed that GSM had a high concentration of total PUFAs (65.17 g/100 g sample), with a high content of ω-6 fatty acids especially linoleic acid (63.63 g/100 g, data not shown). GSM contained also an important amount of fibers (37.76%, data not shown).

### Effects of GSM Diet on Growth Performances and Diarrhea Incidence in DSS-Treated Piglets

After the first DSS challenge, severe diarrhea was observed, in week 2 of the experiment, with 60% of total piglets from the DSS-treated group being affected (Table 2). The incidence of diarrhea in the DSS group was also increased in week 3 of the experiment, after the second DSS challenge, and these piglets remained affected until the end of the experiment (week 4, Table 2). In DSS-treated piglets receiving GSM diet, the diarrhea incidence was below that of the DSS group, throughout the experiment (Table 2).

**Table 2:** Diarrhea incidence in experimental groups.

**Week of experiment**	Experimental group^*^			
	**Control**	**DSS**	**GSM**	**DSS+GSM**
Week 1	16.67	20.00	0.00	40.00
Week 2	33.33	60.00	16.67	40.00
Week 3	16.67	80.00	0.00	0.00
Week 4	0.00	40.00	0.00	20.00

* *Data represent the percentages of diarrhea-affected animals from total number of animals per experimental group (control group: n = 6; DSS group: n = 5; GSM group: n = 6; DSS+GSM group: n = 5), in all the weeks of the experiment*.

There were no significant differences for final body weight, average daily gain, and feed intake between treatments (Table 3). Regarding feed efficiency (FE), our results showed an increased FE in the DSS group (2.12), while both GSM and DSS+GSM groups had similar FE (1.922 and 2.009, respectively), the best FE being observed for the control group (1.797, Table 3). No significant differences in growth and feed intake were found among treatment groups.

**Table 3:** The effect of GSM diet on performance of DSS-treated piglets.

	Experimental group^*^						
	**Control**	**DSS**	**GSM**	**DSS+GSM**	***p*-value**	***p*-value**	***p*-value**
					**(DSS effect)**	**(GSM effect)**	**(DSS × GSM effect)**
ADG (g)	494.1 ± 53.4	435.7 ± 91.0	432.1 ± 23.3	457.1 ± 52	0.693	0.765	0.362
ADFI (g/day/pig)	0.825 ± 0.04	0.925 ± 0.05	0.820 ± 0.04	0.856 ± 0.05	0.358	0.160	0.100
Initial BW (kg)	9.08 ± 0.20	9.00 ± 0.30	9.08 ± 0.30	9.00 ± 0.30	0.943	0.830	0.830
Final BW (kg)	22.92 ± 1.50	21.20 ± 2.40	21.10 ± 0.80	21.80 ± 1.6	0.261	0.922	0.122
FE (feed:gain)	1.797	2.120	1.898	1.873	0.466	0.992	0.889

* *Pigs were fed for 30 days with a control diet or a diet including 8% GSM and challenged or not with DSS. Values are represented as the mean ± SEM (Control group, n = 6; DSS group, n = 5; GSM group, n = 6; DSS + GSM group, n = 5); DSS, dextran sulfate; GSM, grape seed meal; ADFI, average daily feed intake; ADG, average daily gain; FE, feed conversion ratio*.

### Comparison of Richness and Diversity of Gut Microbiota Sequencing

To understand the effect of DSS and GSM on the composition of gut microbiota, we performed 16S rRNA V3–V4 region sequencing. On the whole, 1,111,323 high-quality sequences and 35,981 distinct operational taxonomic units (OTUs) were identified between all the experimental groups from the usable raw data after the optimization process as follows: (1) control group−4807 OTUs; (2) DSS group−4375 OTUs; (3) GSM group−4022 OTUs; and (4) DSS+GSM group−3820 OTUs.

**Figure 1:**
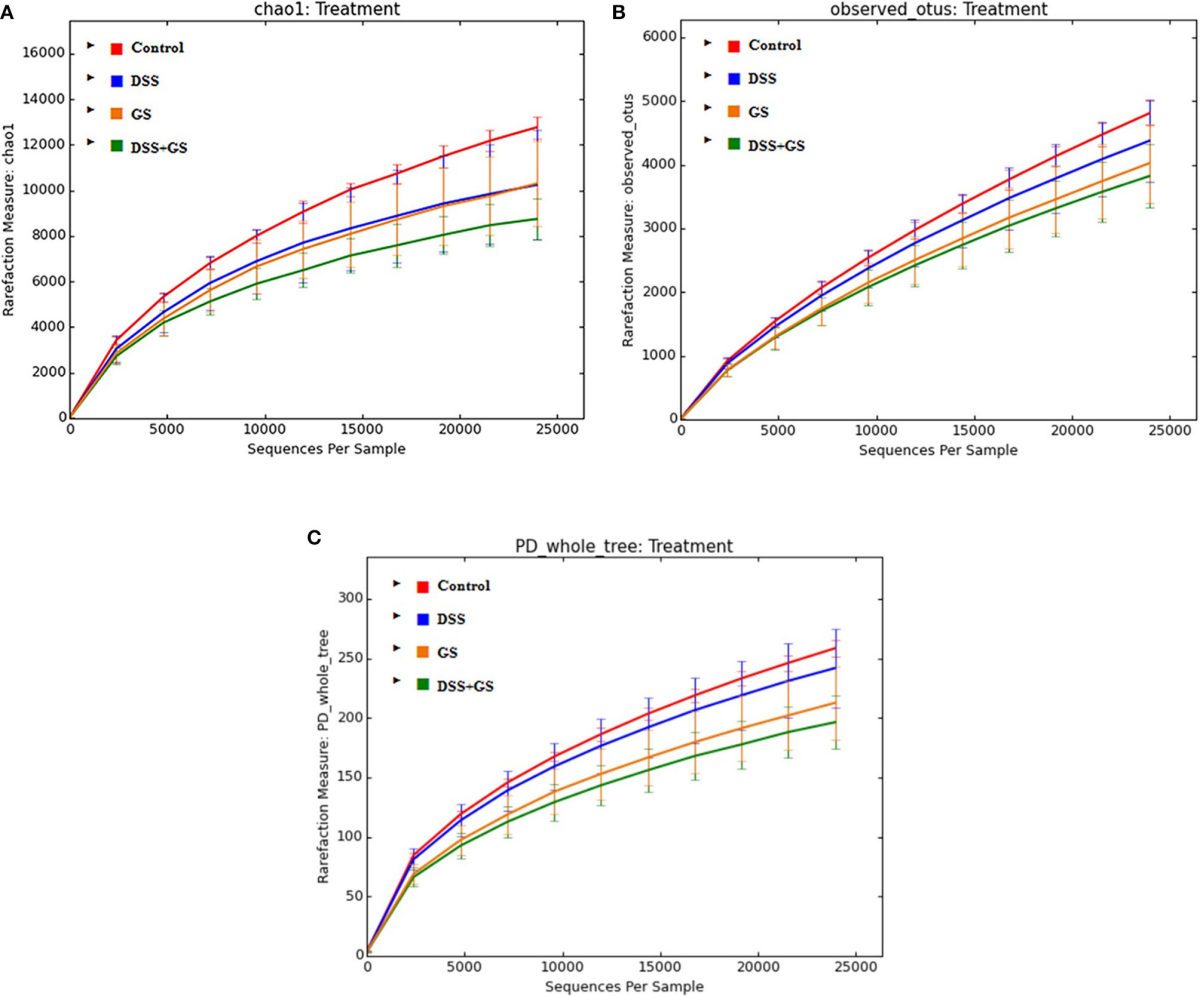
Alpha diversity analysis of dietary groups. The indices are Chao1 **(A)**, observed_otus **(B)**, and PD_whole_tree **(C)**. Control, red; DSS, dextran sodium sulfate, blue; GS, grape seed meal, yellow; DSS+GS, dextran sodium sulfate and grape seed, green.

Based on the sequencing data, the richness of the gut microbiota (Chao1) and the observed OTUs, Chao1, and PD_Whole_Tree indices were decreased after DSS challenge compared to control (Table 4, Figures 1A–C). Similar results were obtained for the GSM group when compared to the control group for all the three indices (Table 4, Figures 1A–C).

**Table 4:** Observed OTUs, PD_Whole_Tree index and Chao 1 mean of the microbiota of piglets treated with DSS and fed with Control or GSM diet.

Experimental group^*^	**Observed OTUs**	**PD_Whole_Tree index**	**Chao1**
Control	4807.2 ± 188^a^	258.5 ± 6^a^	12,766.9 ± 481^a^
DSS	4375.2 ± 651^a,b^	241.9 ± 33^a,b^	10,242.4 ± 407^a,b^
GSM	4022.7 ± 623^a,b^	212.6 ± 30^b^	10,304.8 ± 857^b^
DSS+GSM	3820.9 ± 495^b^	196.4 ± 22^b^	8746.1 ± 901^b^

* *Control and DSS-treated piglets were fed for 30 days with a control diet or a diet containing 8% GSM, as described in the Materials and Methods section. At the end of the experiment, samples of colonic content from all animals (n = 5) were collected and analyzed for identification of microbial groups. Values within a column with different superscript letters are significantly different (p < 0.05)*.

There were significant differences (*p* < 0.05) between the GSM and control groups at the PD_Whole_Tree (212.6 vs. 258.5, Figure 1C) and Chao1 (10304.8 vs. 12766.9, Figure 1A) indices. Also, significant decreased values were found for DSS+GSM compared to the control group for all three indices (Table 4, Figures 1A–C).

**Figure 2:**
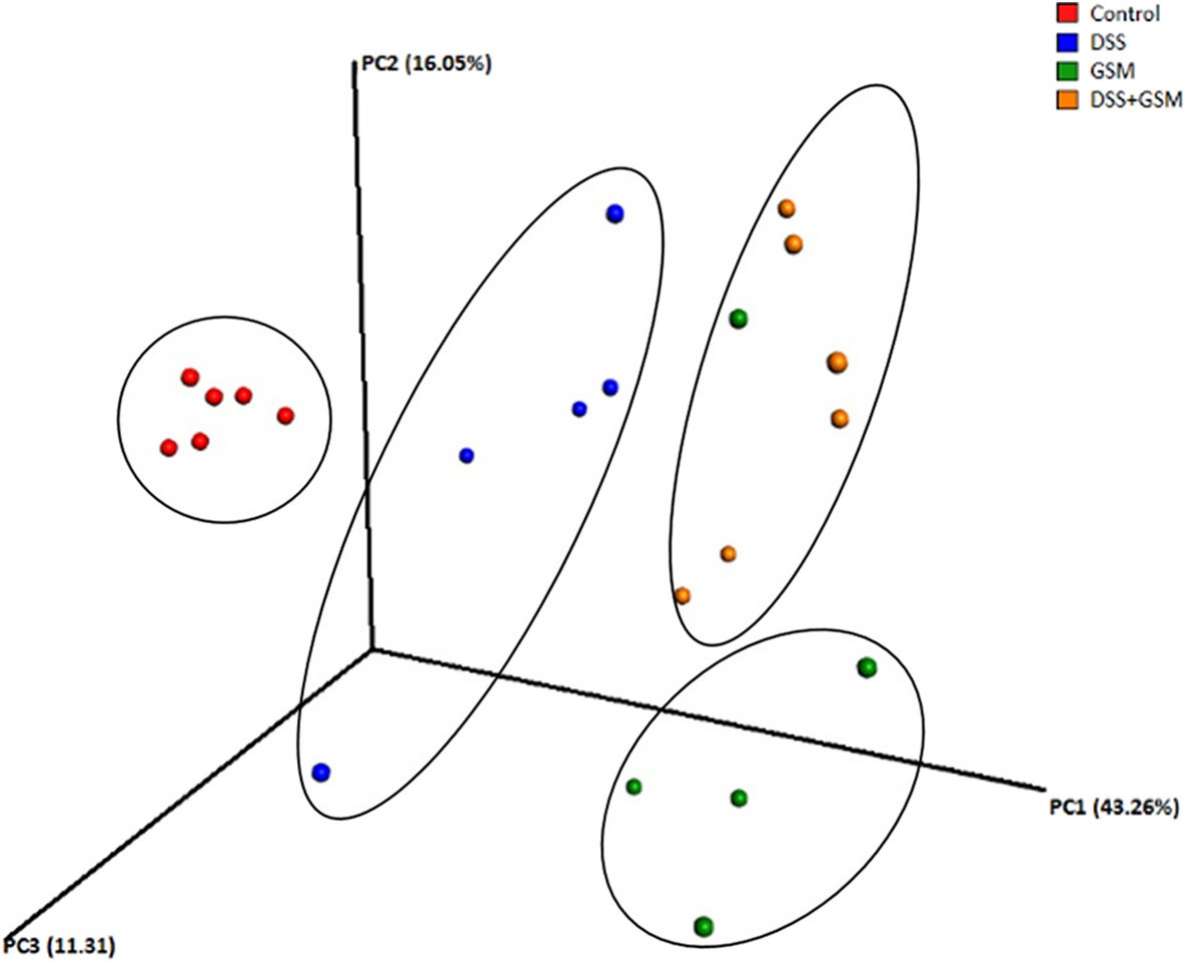
Qualitative principal component analysis based on distance matrix (based on unweighted UniFrac metrics of OTUs). Dietary groups colon piglet samples: control (red), DSS (blue), GSM (yellow), DSS+GSM (green). Ellipses were used to show clustering.

In order to compare the overall microbiota structure, β diversity was analyzed using PCoA (principal coordinate analysis) based on three distance matrices, including Euclidean, unweighted_uniFrac, and weighted_uniFrac (Figure 2).

The four experimental groups used in our study were separated as four clusters along PC1 (43.26%), suggesting that there were significant differences in the dominant bacterial population among the groups (Figure 2).

The results of PCoA showed segregation of samples collected from control and DSS-treated groups especially based on unweighted UniFrac matrix, as demonstrated by the first three principal component scores, which accounted for 43.26%, 16.05%, and 11.31% of total variations.

### Bacterial Phyla Abundances in the Colon of DSS-Treated and GSM Diet-Fed Piglets

**Figure 3:**
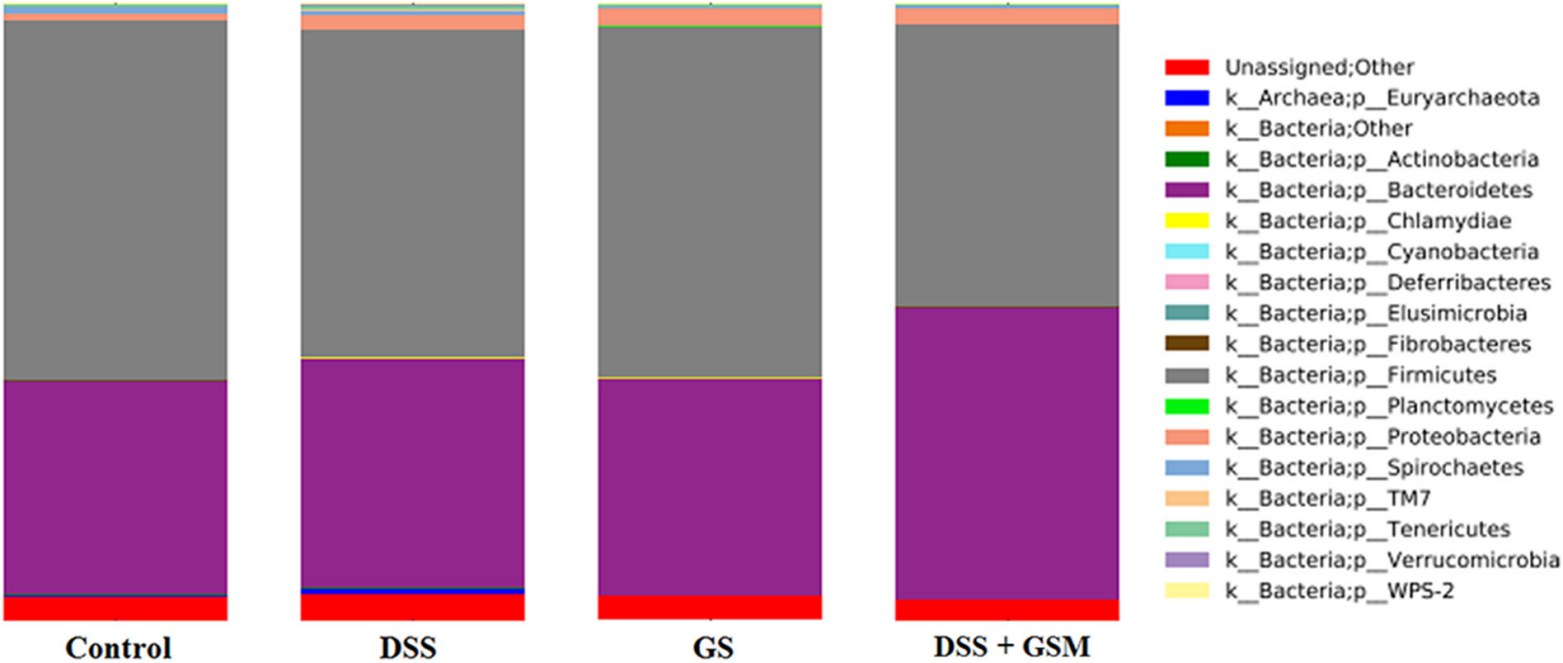
Relative abundance (%) of bacteria phylum as calculated by QIIME. Dietary groups: control, DSS, GSM, and DSS+GSM.

The total sequence reads used in this study were classified into 16 phyla, and one phylum was noted as unassigned. Overall, the bacterial communities were dominated by bacteria belonging to *Firmicutes* (50.5–60.1%), *Bacteroidetes* (36.1–45.8%), and *Proteobacteria* (1.3–3.49%) phyla, whereas a small percentage (0.01–0.09%) belonged to *Spirochaetes, Tenericutes*, and *Euryarchaeota* phyla (Figure 3). The constituent ratios of bacteria at the phylum level were different between DSS-treated and control groups, which was consistent with the results of OTU clustering and PCoA (Figure 3).

**Figure 4:**
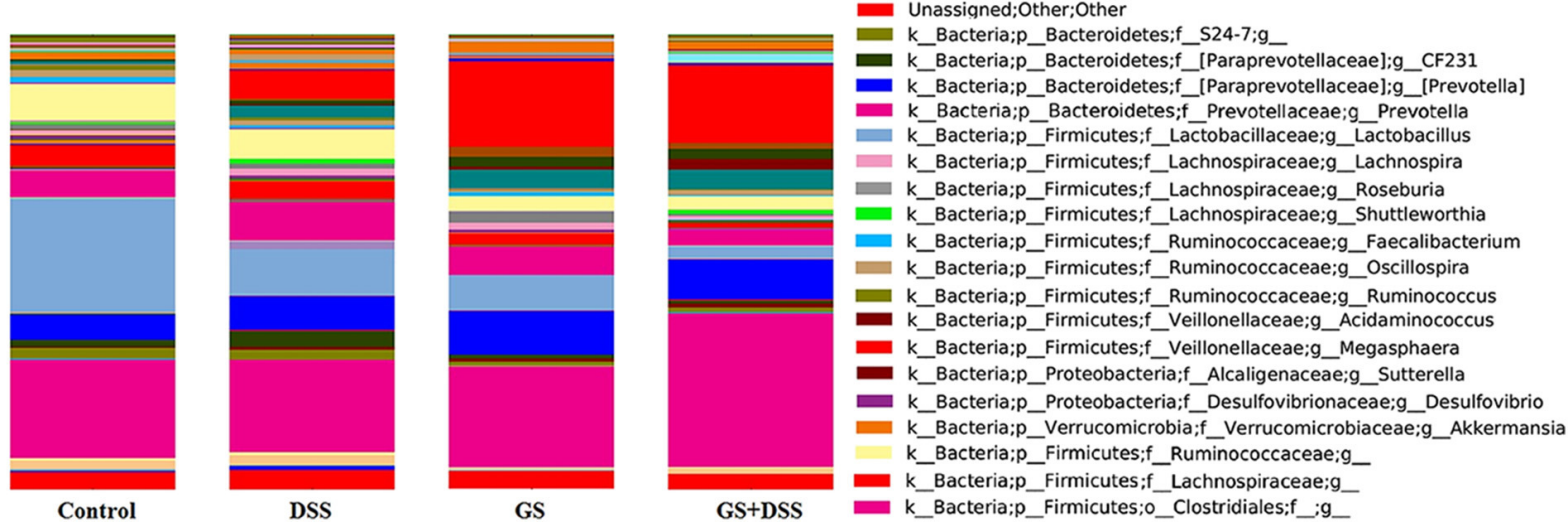
Relative abundances (%) of bacteria genera as calculated by QIIME. Dietary groups: control, DSS, GSM, and DSS+GSM.

Overall, the relative abundance of *Firmicutes* was reduced by the DSS challenge in a significant way (*p* < 0.0001) when compared to the dietary groups (control and GSM group) without DSS challenge (Table 5). The dietary GSM inclusion had a similar relative abundance of *Firmicutes* with the control diet, and in the DSS+GSM group, a slightly lower relative abundance of *Firmicutes* was observed when compared with DSS group alone (50.5 vs. 53.9%, Figure 4) and control (50.5 vs. 60.1).

**Table 5:** Effect of GSM diet on microbiota of DSS-treated piglets (*filum*).

	**Control**	**DSS**	**GSM**	**DSS+GSM**	**DSS effect**			**GSM effect**			**DSS+GSM effect**		
					**η^2^**	***P*-value**	**ω^2^**	**η^2^**	***P*-value**	**ω^2^**	**η^2^**	***P*-value**	**ω^2^**
*Firmicutes (r.a)*	0.60 ± 0.01	0.54 ± 0.01	0.60 ± 0.01	0.50 ± 0.01	0.607	<0.0001^*^	0.578	0.053	0.1026	0.034	0.015	0.373	−0.002
*Bacteroidetes (r.a)*	0.36 ± 0.01	0.38 ± 0.01	0.36 ± 0.01	0.45 ± 0.01	0.375	0.0005^*^	0.347	0.112	0.0332^*^	0.089	0.133	0.0217^*^	0.109
*Proteobacteria (r.a.)*	0.01 ± 0.00	0.03 ± 0.00	0.03 ± 0.00	0.02 ± 0.00	0.094	0.0032^*^	0.085	0.065	0.0108	0.057	0.693	<0.0001^*^	0.679
*Lactobacillus (r.a.)*	0.30 ± 0.02	0.12 ± 0.01	0.09 ± 0.01	0.03 ± 0.00	0.285	<0.0001^*^	0.277	0.535	<0.0001^*^	0.526	0.074	0.0023^*^	0.068
*Prevotella (r.a.)*	0.23 ± 0.01	0.21 ± 0.02	0.22 ± 0.02	0.33 ± 0.00	0.118	0.0378^*^	0.093	0.197	0.0096^*^	0.17	0.258	0.0039^*^	0.23
*Megasphaera (r.a.)*	0.0003 ± 0.00	0.068 ± 0.00	0.222 ± 0.00	0.211 ± 0.02	0.021	0.0414^*^	0.016	0.864	<0.0001^*^	0.856	0.038	0.0076^*^	0.034
*CF231 (r.a.)*	0.016 ± 0.00	0.034 ± 0.00	0.01 ± 0.00	0.007 ± 0.0	0.115	0.0008^*^	0.107	0.543	<0.0001^*^	0.532	0.215	<0.0001^*^	0.207
*Anaerovibrio (r.a.)*	0.004 ± 0.00	0.0073 ± 0.00	0.03 ± 0.00	0.027 ± 0.0	0.00002	0.9769	−0.02	0.553	0.0002^*^	0.516	0.007	0.6025	−0.017
*Roseburia (r.a)*	0.015 ± 0.00	0.027 ± 0.00	0.005 ± 0.00	0.013 ± 0.00	0.339	<0.0001^*^	0.328	0.49	<0.0001^*^	0.477	0.004	0.2529	0.004

*r.a., relative abundance; η^2^, Eta squared; ω^2^, Omega squared; Microbiota (filum) relative abundance values are represented as mean with their standard errors. ANOVA (two-way) was performed to analyse the main factors DSS, GSM, and DSS × GSM interaction effects on microbiota incidence. Calculation and effect size magnitudes are given in Materials and Methods section*.

* *P-values lower than 0.05 are statistically significant*.

The *Bacteroidetes* phylum increased significantly (*p* = 0.0005) in relative abundance under the effect of DSS as well as under the effect of GSM but to a lesser extent (*p* = 0.0332, η^2^ 0.11 vs. 0.375, 45.8%, Table 5). The *Proteobacteria* relative abundance was also influenced by DSS challenge as well as the GSM treatment increasing significantly (*p* = 0.0032 for DSS effect and 0.0108 for GSM, Table 5). In the interaction between DSS and GSM, a lower relative abundance was observed for the *Proteobacteria* phylum.

An interesting aspect of the dietary treatments was that the *Bacteroidetes/Firmicutes* observed ratio tended to increase in the DSS group (0.71) and reached the highest value in the DSS+GSM group (0.90) when compared to the other groups. This ratio was similar in both control and GSM dietary groups (0.60 and 0.61).

### Microbial Genus Relative Abundances in Gut of DSS-Treated and GSM Diet-Fed Piglets

One hundred forty-nine bacterial taxa were identified as the most frequent species among the groups. Of these, 85 were identified at the genus level, and the remaining 64 could only be classified at the level of family or order taxon.

At the genus level, *Lactobacillus, Prevotella*, and *Megasphaera* dominated the colon microbiota among the four dietary groups while genus like *CF231* (a member of *Paraprevotellaceae* family), *Anaerovibrio*, and *Roseburia* have a lower abundance (lower than 4%, Figure 4). The highest *Lactobacillus* relative abundance was noticed in the dietary groups not affected by either DSS or GSM, and it was lowered in a significant way (*p* < 0.0001) in the dietary groups affected by DSS or GSM (Figure 4 and Table 6).

**Table 6:** Effect of GSM diet on microbiota of DSS-treated piglets (*genus*).

	**Control**	**DSS**	**GSM**	**DSS+GSM**	**DSS effect**			**GSM effect**			**DSS+GSM effect**		
					**η^2^**	***P*-value**	**ω^2^**	**η^2^**	***P*-value**	**ω^2^**	**η^2^**	***P*-value**	**ω^2^**
*Lactobacillus (r.a.)*	0.30 ± 0.02	0.12 ± 0.01	0.09 ± 0.01	0.03 ± 0.00	0.285	<0.0001^*^	0.277	0.535	<0.0001^*^	0.526	0.074	0.0023^*^	0.068
*Prevotella (r.a.)*	0.23 ± 0.01	0.21 ± 0.02	0.22 ± 0.02	0.33 ± 0.00	0.118	0.0378^*^	0.093	0.197	0.0096^*^	0.17	0.258	0.0039^*^	0.23
*Megasphaera (r.a.)*	0.0003 ± 0.00	0.068 ± 0.00	0.222 ± 0.00	0.211 ± 0.02	0.021	0.0414^*^	0.016	0.864	<0.0001^*^	0.856	0.038	0.0076^*^	0.034
*CF231 (r.a.)*	0.016 ± 0.00	0.034 ± 0.00	0.01 ± 0.00	0.007 ± 0.0	0.115	0.0008^*^	0.107	0.543	<0.0001^*^	0.532	0.215	<0.0001^*^	0.207
*Anaerovibrio (r.a.)*	0.004 ± 0.00	0.0073 ± 0.00	0.03 ± 0.00	0.027 ± 0.0	0.00002	0.9769	−0.02	0.553	0.0002^*^	0.516	0.007	0.6025	−0.017
*Roseburia (r.a)*	0.015 ± 0.00	0.027 ± 0.00	0.005 ± 0.00	0.013 ± 0.00	0.339	<0.0001^*^	0.328	0.49	<0.0001^*^	0.477	0.004	0.2529	0.004

*r.a., relative abundance; η^2^, Eta squared; ω^2^, Omega squared; Microbiota (filum) relative abundance values are represented as mean with their standard errors. ANOVA (two-way) was performed to analyse the main factors DSS, GSM, and DSS × GSM interaction effects on microbiota incidence. Calculation and effect size magnitudes are given in Materials and Methods section*.

* *P-values lower than 0.05 are statistically significant*.

For *Prevotella* genus, the dietary GSM inclusion had a significant (*p* = 0.0096) positive effect on its relative abundance. Also, noticeable differences were observed in the interaction between the DSS and GSM with the highest effect size (η^2^ = 0.25, Figure 4 and Table 6). The *Megasphaera* genus relative abundance was stimulated by the DSS and GSM effect in a significant proportion (*p* = 0.0076, η = 0.038). On the *CF231* genus, DSS had a significant effect on stimulating the bacterial abundance (*p* = 0.0008). The addition of the GSM lowered the relative abundance count significantly (*p* < 0.0001) and in a sizeable way (η^2^ = 0.543) in a manner as to modulate the effect of DSS (Figure 4 and Table 6).

The *Anaerovibrio* genus was influenced significantly (*p* = 0.0002) only by the GSM diet alone, the effect size being noticeable when compared to the DSS effect (Table 6). DSS challenge also significantly increased the relative abundance of bacteria from *Roseburia* genus (*p* < 0.0001) being in contrast with the effect of the GSM diet, which acted by inhibiting the *Roseburia* genus (*p* = 0.0001, η^2^ = 0.5, Table 6).

**Figure 5:**
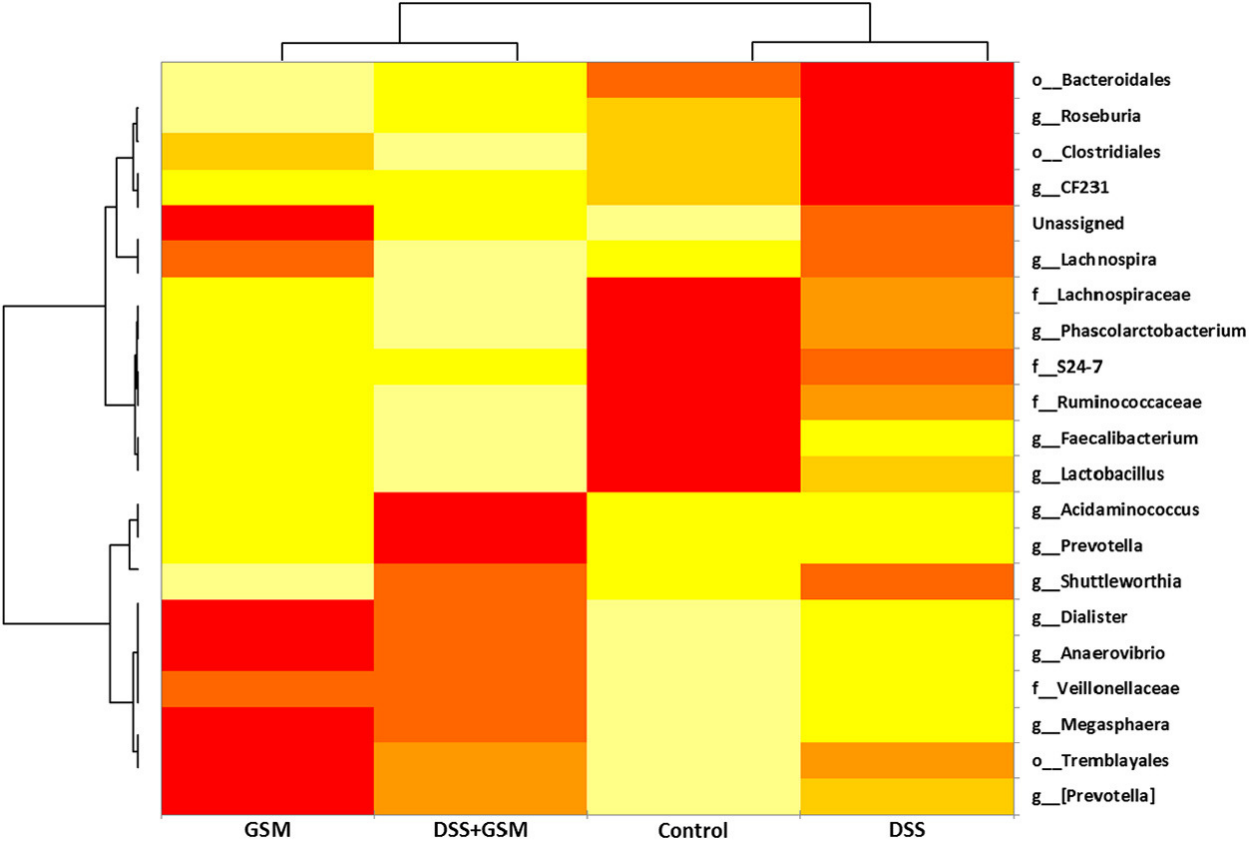
Heatmap of the most abundant genus, family, and order, based on the dietary groups. Clustering between groups and also between taxa was selected. The relative abundance is colored in shades of yellow (low relative abundance) to red (high relative abundance).

To have a comprehensive image on the dynamics and influence of the DSS and GSM treatments on the microbiota (especially on the most abundant species), we used comparative analysis at the genus level, as shown in the heatmap presented in Figure 5.

The higher the abundance of an OTU in a sample, the more intense is the red color at the corresponding position in the heatmap. By default, the OTUs (rows) were clustered by QIIME, and the samples (columns) were presented in the order in which they appear in the OTU table. When observed at the family taxa, a downward trend can be seen for the *Lactobacillaceae, Ruminococcaceae*, and *Lachnospiraceae* bacterial families, from control to DSS and DSS+GSM groups while being progressively replaced by *Prevotellaceae* and *Veillonellaceae* families, respectively, for the same dietary groups (Figure 5). The GSM group was characterized by a high abundance of *Anaerovibrio, Megasphera*, and *Trembyales* and a lower abundance of *Roseburia, CF231, Fecalibacterium, Lactobacillus*, and *Prevotella*. The genera *Dialister, Acidaminococcus*, and *Faecalibacterium* were also encountered in the colon content of the piglets but in a lower percentage (Figure 5).

### The Effects of the DSS and GSM Diet on the Fecal SCFA Production

The effects of DSS challenge and GSM diet on SCFA production by anaerobic bacteria are presented in Table 7. Although there were no statistical differences in the total SCFA concentration between the experimental groups, statistical differences in concentration were observed in the case of some SCFA.

**Table 7:** The composition in SCFAs of colon content collected from piglets.

**Analyte (SCFAs)**	Experimental group^*^						
	**Control**	**DSS**	**GSM**	**DSS+GSM**	***p*-value (DSS effect)**	***p*-value (GSM effect)**	***p*-value (DSS × GSM effect)**
Total SCFA (mM/g sample)	13.3 ± 2.3	15.8 ± 1.2	12.6 ± 0.5	14.8 ± 0.8	0.060	0.594	0.972
Acetate (%)	53.7 ± 1.8^a^	52.1 ± 1.2^a^	48.1 ± 2.2^a^	42.5 ± 1.1^b^	0.066	<0.0001	0.386
Propionate (%)	26.1 ± 0.5^b^	26.3 ± 1.6^b^	25.0 ± 1.0^b^	29.0 ± 0.9^a^	0.058	0.314	0.044
Isobutyrate (%)	1.9 ± 0.3^b^	2.4 ± 0.3^b^	2.7 ± 0.2^a^	2.5 ± 0.3^a^	0.851	0.051	0.160
Butyrate (%)	13.0 ± 0.9^b^	12.1 ± 0.3^b^	16.6 ± 1.0^a^	16.4 ± 0.3^a^	0.605	<0.0001	0.898
Isovalerate (%)	1.6 ± 0.2	2.7 ± 0.6	2.2 ± 0.4	1.9 ± 0.6	0.285	0.842	0.099
Valerate (%)	3.6 ± 0.4^b^	4.4 ± 0.3^b^	5.3 ± 0.5^a^	7.6 ± 0.4^a^	0.002	<0.0001	0.075

* *Control and DSS-treated piglets were fed for 30 days with a control diet or a diet containing 8% GSM, as described in Materials and Methods section. At the end of the experiment, samples of colonic content from all animals (n = 5) were collected and analyzed for the composition of SCFAs. Values within a row with different superscript letters are significantly different (p < 0.05)*.

The acetate proportion was significantly lowered with the addition of GSM (*p* < 0.0001). The effect of the GSM was also felt at the butyrate percentage, it being significantly higher (*p* < 0.0001) compared to control and DSS group. The percentage of valerate was also increased by both the challenge of GSM and that of DSS. The interaction between the effects only seems to affect the propionate levels (*p* = 0.044).

## Discussion

The IBD presents a worldwide health concern because of the lack of a cure and definitive therapies to tackle the issue (1, 32). The aim of the present study was to analyze and discuss if nutritional interventions based on bioactive compounds from grape seed could ameliorate and change the microbial composition affected by-product through induced IBD by using the pig as an animal model.

Medication alternatives in IBD such as polyphenolic compounds (17, 33, 34), SCFAs, and PUFAs (35) have been studied lately by many research groups with promising results. The biologic activity and underlying mechanisms have rarely been identified (36). However, Bousenna et al. (17) evaluated a polyphenol-rich grape pomace extract on rats challenged with DSS and observed attenuation in the clinical signs, colon shortening, and limitation of histological lesions usually observed in DSS-induced colon inflammation. Another study carried out by Aboura et al. (37) reported that polyphenol-rich infusion from carob leaves and Opuntia cladodes presented anti-inflammatory effects, counteracted intestinal permeability and colon histological lesions, and decreased DSS-induced pro-inflammatory cytokine expression in mice. Samsami-Kor et al. (38) also showed that resveratrol, a highly studied polyphenol that is abundant in natural sources like grapes, could decrease clinical disease activity index and quality of life in patients with ulcerative colitis (UC) in a randomized, double-blind clinical trial. Increasing SCFA metabolites in the colon via administration of prebiotic high-fiber diets in combination with probiotic bacteria (*Lactobacillus, Bifidobacterium*, and *Faecalibacterium*) were also studied for intestinal lesion amelioration, gut barrier improvement, anti-inflammatory effect, and as preventive strategies in the management of DSS in mice (39–41). Short-term supplementation with eicosatetraenoic (n-3) free fatty acid was evaluated in a study by Prossomariti et al. (42), improving endoscopic and histological inflammation while also modulating microbiota composition in long-standing UC patients. In both animal and human, gut microbiota participated in different host processes, and an imbalance in its ecological composition may cause systemic and intestinal dysfunction (43). Modulation or aberrations in the gut microbial community have been shown in IBD. The dysbiosis effects associated with IBD have been characterized usually as a perturbation in the ratio of *Bacteroidetes* over *Firmicutes* (44). Modifications of the microbiota at the phylum, genus, and species level are known to occur when DSS is used to induce inflammation (45). Overall, the *Bacteroidetes/Firmicutes* ratios in DSS-afflicted groups were found to be higher than those of the control and associated with dysbiosis (9, 44, 46, 47). In the present study, dramatic changes in overall ratio and diversity of the gut microbiota were observed in DSS-treated pigs receiving GSM diet when compared to the control and other groups. Thus, the ratio of *Bacteroidetes/Firmicutes* significantly increased (*p* < 0.05) in the DSS-challenged group, whereas no difference between GSM and control diet was observed. The administration of the GSM diet to pigs treated with DSS was not able to decrease the *Bacteroidetes/Firmicutes* ratio, which remains higher in comparison with the control.

An increase in the relative abundances of the *Bacteroidetes* phylum and *Bacteroidales* and *Clostridiales* orders and an overall decrease in *Firmicutes* phylum were found by Imhann et al. (48), which were linked to irritable bowel diseases. Similarly, herein, the *Firmicutes* phylum was decreased in a significant way (*p* < 0.05) under DSS effect and was not affected by the addition of GSM into the diet in a significant way. *Clostridium, Roseburia, Acidaminococcus*, and *Escherichia* are often cited as the genera usually found in abundance in irritable bowel diseases (48–52) and DSS treatment, while *Lactobacillus, Prevotella*, and *Faecalibacterium* are cited as negatively impacted or inversely correlated with the severity of the disease (33, 53–56). Indeed, in our work, *Roseburia, Megasphera*, and *CF231* increased significantly under the DSS presence while *Lactobacillus* registered a significant decrease overall. Interesting is that GSM and especially DSS+GSM treatment progressively decreased the relative abundance of *Lactobacillus*. Dietary GSM was able to counteract the abundance of *Roseburia*, linked in other studies to an increase in abundance and for its role in the onset and progression of IBD dysbiosis (49, 50, 57). The GSM diet also modulated the abundances of *Anaerovibrio* (increasing) and *CF231* (decreasing), which play essential roles in the repair of the intestinal epithelial damage and are constituents of the gut microbiota core (58). Interestingly, the GSM diet alone determined a significant decrease of *Lactobacillus* spp. in comparison with all the other diets, including control; moreover, the DSS-treated pigs that received dietary GSM registered the lowest *Lactobacillus* abundance. Generally, *Lactobacillus* spp. are associated with positive effects in the large intestine, being able to enhance the gut barrier functions, to modulate the immune system, and to compete with pathogen species for the large intestine colonic mucosa (59). The observed reduction in the abundance of *Lactobacillus* spp. in pigs with DSS-induced inflammation or pigs with an intake of GSM diet and dramatically in DSS+GSM groups might be associated with the negative impact that the DSS has on this genus (55) as well as the interaction between *Lactobacillus*, the type of phenolic compounds and their concentration as described in the scientific literature (56, 60). However, the findings are controversial. For example, Ozdal et al. (61) found an increase in *Lactobacillus* abundance with gallic acid, punicalagin, proanthocyanidins, and resveratrol and no effect with (+)-catechin, (–)-epicatechin, and quercetin, while Pastorkova et al. (62), investigating the antimicrobial potential of 15 grape phenolic compounds against yeast and acetic acid bacteria from wine, found that resveratrol, pterostilbene, and luteolin presented the highest antibacterial effect. The grape seed extract was also shown to inhibit the growth of *Streptococcus thermophilus, Bifidobacterium lacticus, Lactobacillus fermentum*, and *acidophilus* due to their perceived sensitivity to the polyphenol fraction flavan-3-ols (63). Nevertheless, the activity of some biological compounds could be masked by that of others, which constrain the understanding of the exact synergistic effect that could happen (56). In contrast, a significantly higher level of *Prevotella* belonging to *Bacteroidetes* phylum was observed in our study being stimulated by the interaction between DSS and GSM. *Prevotella* genus is considered a commensal, with essential functions in maintaining the gut health of pigs due to its frequent occurrence in the healthy pig gut microbiota, its rare involvement in bacterial infection, and the high butyrate synthesis (64). The involvement of *Prevotella* in the fermentation of plant-derived non-digestible fibers to SCFAs has been observed in piglets (65), allowing them to adapt to new dietary conditions. In human, *Prevotella* has been related to diets rich in vegetables and fruits like vegetarian and Mediterranean diets (66). De Cruz et al. and Slifierz et al. have found interesting results linking the presence of *Prevotella* with remission in Crohn's disease and recovery from chronic effects of DSS-induced colitis (53, 54). Herein, piglets subjected to DSS-induced inflammation (DSS+GSM group) consuming the GSM diet had a higher *Prevotella* in the colon.

GSM effect and DSS challenge alone and in interaction caused a significant (*p* < 0.0039) increase in *Megasphaera* genus abundance. *Megasphaera* including the lactate-utilizing bacteria represents the healthy microbiota of pigs, which maintain the pH balance and play an essential role in the fermentation of a variable part of dl-lactate to butyrate, with some of the highest concentrations of butyrate in comparison to other anaerobic butyrate-producing bacteria belonging to the *Firmicutes* (67). Other studies have also pointed on the beneficial effects of *Lactobacillus, Megasphaera*, and their complementarity and association in the production of SCFAs and in promoting intestinal health in pigs (54, 68).

SCFAs produced primarily from the microbial fermentation of dietary fiber appear to be critical mediators of the beneficial effects elicited by the gut microbiome (69). GSM, a by-product of the grape seed oil process, is mainly composed of dietary fibers and polyphenols that offer an ideal substrate for colonic bacteria in their process of colonic fermentation, thus increasing the concentration levels of SCFAs. Indeed, the colonic concentration of butyrate was increased by the GSM diet in both GSM and DSS+GSM groups when compared to control or DSS groups indicative of an increased beneficial microbial activity and a modulatory effect of the GSM. Among the SCFAs, butyrate in particular has been shown to promote commensal bacterial growth (70), provide an energy source for epithelial cells of the host (71), and enhance the overall gut barrier integrity (72–76). A high propionate level was also achieved in the DSS+GSM group comparatively to the rest, which is associated with an overall amelioration and improvement of intestinal barrier function (77).

The bacteria from the phylum *Bacteroidetes* are typically associated with the production of acetate and propionate while the *Firmicutes* phylum [*Megasphaera, Faecalibacterium* (*Prevotella*)] mainly produce butyrates (78). The significantly high levels of butyrate observed from GSM and GSM+DSS were highly correlated with the same species as *Megasphaera, Faecalibacterium*, and *[Prevotella]* while the high propionate and acetate concentrations from the GSM+DSS dietary group were also correlated with members of the Firmicutes phylum (*Roseburia* for propionate and *Shuttleworthia* for acetate). Our results further corroborate with similar findings of other studies that place *Megasphera [Prevotella]*, and *Faecalibacterium* as the most important producers of SCFAs (78). SCFA results confirm the fact that the GSM diet with the high-fiber content contributes, through microbial fermentation, to the significant production of SCFAs with a demonstrated effect on intestinal bowel diseases. In accordance with literature data (11, 79), GSM also provides an excellent matrix for their polyphenol, fiber-rich content, which can further contribute to the amelioration of DSS-induced UC effects by their increased anti-inflammatory and antioxidant capacity, thus improving gastrointestinal health (11, 79).

GSM with high-fiber, -polyphenol, and -PUFA content increased the production of butyrate and isobutyrate, stimulated the growth of beneficial genera like *Prevotella* and *Megasphaera*, while countering the relative abundance of *Roseburia*, reducing it to half of the DSS value and contributing to the management of the DSS effects.

## Conclusions

Our results showed that GSM, a common by-product of grape seed oil processing, which contains significant concentrations of several bioactive compounds, like polyphenols, PUFA, fibers, minerals, etc., had a selective modulatory effect on several bacterial genera in the colon of pigs challenged with DSS. Our study demonstrated that *Bacteroidetes* and *Firmicutes* phyla were the most prevalent bacteria in the colon of pig irrespective of the treatment. DSS challenge affected the colonic bacteria, increasing overall the abundance of *Proteobacteria* phylum and of *Roseburia*, associated with the progression of IBD, and affected the *Bacteroidetes/Firmicutes* ratio contributing to an overall loss in the microbiota species diversity. GSM increased the production of butyrate and isobutyrate in pigs receiving dietary GSM and stimulated the growth of beneficial genera like *Prevotella* and *Megasphaera* while reducing to half the relative abundance of *Roseburia* registered in the DSS dietary group. GSM is an available raw material source of bioactive compounds that might be used as supplement functional food in IBD. For practical applicability, this dry form of grape seed (meal) could be quickly processed by encapsulation and served along with the daily diet. However, further researches testing other GSM dietary concentrations and their effects are necessary.

## Data Availability Statement

The datasets generated for this study can be found in the ENA database (https://www.ebi.ac.uk/ena/browser/home) under the name PRJEB34923 (ERP117900) | Raw sequences 16s.

## Ethics Statement

The procedures were in agreement with the Romanian Law 206/2004 and the EU Directive 98/58/EC for handling and protection of animals used for experimental purposes. The experimental protocol was approved by the Ethical Committee of the National Research-Development Institute for Animal Nutrition and Biology, Baloteşti, Romania (Ethical Committee no. 52/2014).

## Author Contributions

DM, GP, and IŢ realized the design of the experiment. IG and GP performed the DNA extraction. IG analyzed the raw microbiota data. LP and IG performed the statistical analysis. AC analyzed the SCFA content in colon samples. IG, GP, and IŢ wrote the manuscript.

### Conflict of Interest

The authors declare that the research was conducted in the absence of any commercial or financial relationships that could be construed as a potential conflict of interest.
